# Surgical Simulations Based on Limited Quantitative Data: Understanding How Musculoskeletal Models Can Be Used to Predict Moment Arms and Guide Experimental Design

**DOI:** 10.1371/journal.pone.0157346

**Published:** 2016-06-16

**Authors:** Jennifer A. Nichols, Michael S. Bednar, Wendy M. Murray

**Affiliations:** 1 Department of Biomedical Engineering, Northwestern University, Evanston, Illinois, United States of America; 2 Departments of Physical Medicine & Rehabilitation and Physical Therapy & Human Movement Sciences, Northwestern University Feinberg School of Medicine, Chicago, Illinois, United States of America; 3 Sensory Motor Performance Program, Rehabilitation Institute of Chicago, Chicago, Illinois, United States of America; 4 Edward Hines Jr. VA Hospital, Hines, Illinois, United States of America; 5 Department of Orthopaedic Surgery and Rehabilitation, Stritch School of Medicine, Loyola University–Chicago, Maywood, Illinois, United States of America; Georgia State University, UNITED STATES

## Abstract

The utility of biomechanical models and simulations to examine clinical problems is currently limited by the need for extensive amounts of experimental data describing how a given procedure or disease affects the musculoskeletal system. Methods capable of predicting how individual biomechanical parameters are altered by surgery are necessary for the efficient development of surgical simulations. In this study, we evaluate to what extent models based on limited amounts of quantitative data can be used to predict how surgery influences muscle moment arms, a critical parameter that defines how muscle force is transformed into joint torque. We specifically examine proximal row carpectomy and scaphoid-excision four-corner fusion, two common surgeries to treat wrist osteoarthritis. Using models of these surgeries, which are based on limited data and many assumptions, we perform simulations to formulate a hypothesis regarding how these wrist surgeries influence muscle moment arms. Importantly, the hypothesis is based on analysis of only the primary wrist muscles. We then test the simulation-based hypothesis using a cadaveric experiment that measures moment arms of both the primary wrist and extrinsic thumb muscles. The measured moment arms of the primary wrist muscles are used to verify the hypothesis, while those of the extrinsic thumb muscles are used as cross-validation to test whether the hypothesis is generalizable. The moment arms estimated by the models and measured in the cadaveric experiment both indicate that a critical difference between the surgeries is how they alter radial-ulnar deviation versus flexion-extension moment arms at the wrist. Thus, our results demonstrate that models based on limited quantitative data can provide novel insights. This work also highlights that synergistically utilizing simulation and experimental methods can aid the design of experiments and make it possible to test the predictive limits of current computer simulation techniques.

## Introduction

Orthopaedic surgery imposes substantial geometric changes on the musculoskeletal system. Changing geometry directly affects joint congruence and muscle-tendon paths, which in turn affect joint mechanics, muscle-tendon force-generating parameters, and even post-operative functional outcomes. Ideally, the geometric changes imposed by surgery could be input into biomechanical models, the models would appropriately calculate any changes to the joints and muscles, and computer simulations could then be used to predict functional outcomes. Accurate, predictive simulations would improve treatment decisions by allowing the comparison of multiple surgical procedures and providing insights into the procedures with the best outcomes.

To date, biomechanical models and simulations have been successfully used to investigate a wide variety of orthopaedic problems, including tendon transfers [1–4], nerve transfers [5], osteoarthritis [6–8], and ligament injuries [9–11]. The status quo research paradigm for these investigations includes: first, gathering experimental data to quantitatively describe biomechanical parameters specific to a given clinical condition (e.g., joint kinematics [12–14], muscle moment arms [15, 16], muscle architecture parameters [17–20]); then, incorporating these data into biomechanical models representing the given condition (e.g., [21, 22]); and finally, performing simulations to examine how the biomechanical parameters influence outcomes of broad clinical interest (e.g. strength [21, 23] or the ability to perform activities of daily living [24, 25]). The current simulation-based research paradigm has made it possible to study complex research questions that are nearly impossible to investigate in a laboratory setting. For example, simulation studies can accurately estimate difficult to measure parameters, such as joint loads (e.g., [7, 24, 26, 27]), or systematically evaluate whether outcomes are sensitive to changes in individual (or combinations of) biomechanical parameters (e.g., [3, 23]). The utility of biomechanical simulations to study clinical problems is well illustrated by the publication of simulation studies in both scientific (e.g., [10, 11, 21, 23]) and surgical journals (e.g., [4, 5, 27, 28]).

Despite the widespread adoption and application of biomechanical simulations, the ability to use the current simulation research paradigm to solve clinical problems is highly dependent on the availability, cost, and ease of obtaining the experimental data necessary to build and validate surgical simulations. Importantly, in the current paradigm, building and validating biomechanical models of a given clinical condition is the rate-limiting step. The most widely used rigid-body musculoskeletal models, which describe asymptomatic individuals, leverage decades of experimental work (e.g., [29, 30]). The general impact of biomechanical simulation would be greater if the parameters underlying those simulations could be accurately predicted based on limited quantitative data, thereby eliminating the need for time-consuming experiments and streamlining the development of surgical simulations. The need for experimental data is particularly problematic when studying orthopaedic procedures because the necessary data simply do not exist. For example, a critical biomechanical parameter that is explicitly determined by geometry is moment arm, which transforms the force an individual muscle develops into the torque it generates about a joint. Yet, the influence of orthopaedic procedures on muscle moment arms is rarely investigated. There are over 500 ICD-9 codes classifying distinct diseases and procedures in the upper limb [31]. Yet, moment arms have been measured following less than 20 procedures in the shoulder [28, 32–38], elbow [39], forearm [40, 41], wrist [42–47], and hand [48–50]. The lack of studies measuring moment arms following orthopaedic procedures highlights that performing these experiments is difficult; they require a specific type of research environment that includes scientists skilled at collecting moment arm data as well as surgeons available to properly perform the surgical procedure under investigation. Thus, predicting moment arms based on only the known geometric changes imposed by surgery would represent a step toward transforming the field of biomechanical modeling from one impeded by tedious experiments to one empowered by predictive capability. Predicting muscle moment arms, however, is challenging because surgically imposed geometric changes do not just influence moment arm, but also influence the underlying muscle mechanics and joint kinematics that define moment arms. This means that to use a surgical model to estimate moment arms requires also estimating the parameters on which moment arms depend, namely muscle lines of action and joint axes of rotation. To what extent moment arms can be estimated without data describing these parameters is unknown.

**Fig 1:**
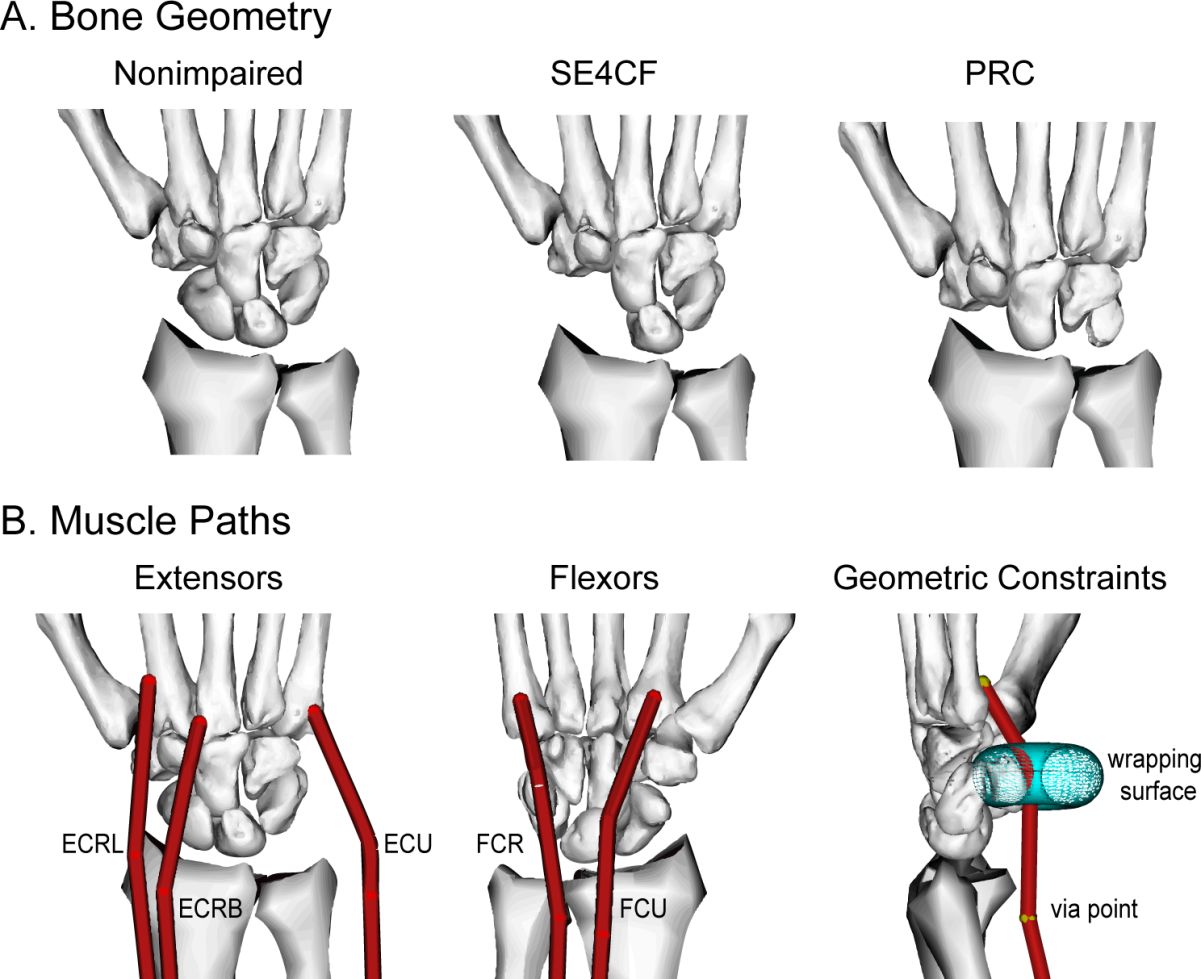
Wrist Models. (A) Bone geometry and (B) muscle paths implemented in the nonimpaired, SE4CF, and PRC models. Only the five primary wrist muscles were included: *extensor carpi radialis longus* (ECRL), *extensor carpi radiali brevis* (ECRB), *extensor carpi ulnaris* (ECU), *flexor carpi radialis* (FCR), and *flexor carpi ulnaris* (FCU). Muscle paths were constrained to anatomically realistic lines of action using via points and wrapping surfaces.

This study aims to challenge the status quo research paradigm by evaluating whether models based on the limited quantitative data describing joint kinematics, that are available in the published literature, can predict changes in moment arms. We specifically examine two common wrist surgeries, proximal row carpectomy (PRC) and scaphoid-excision four-corner fusion (SE4CF). These surgeries were studied because they are used to treat the same degenerative conditions (e.g., osteoarthritis), but each imposes substantial changes to the wrist’s geometry. Importantly, the imposed geometric changes are very different between the two procedures (Fig 1A). Thus, it seems intuitive that a critical difference between these procedures is how the imposed geometric changes influence muscle moment arms. However, the data necessary to estimate moment arms (i.e., axes of rotation and muscle lines of action) following these procedures is not fully known. Wrist axes of rotation have not been reported following SE4CF, and have been reported in only one study following PRC [12]. In general, how the changes in skeletal geometry imposed by orthopaedic surgery influence muscle-tendon paths is not fully understood. As a result, we developed multiple models of PRC and SE4CF, based on a range of reasonable assumptions regarding joint axes of rotation and muscle-tendon paths. We utilized these models to develop a general hypothesis regarding how the two surgeries influence muscle moment arms. We then performed PRC and SE4CF in cadaveric specimens to measure moment arms and evaluated the veracity of our simulation-based hypothesis.

## Methods

**Fig 2:**
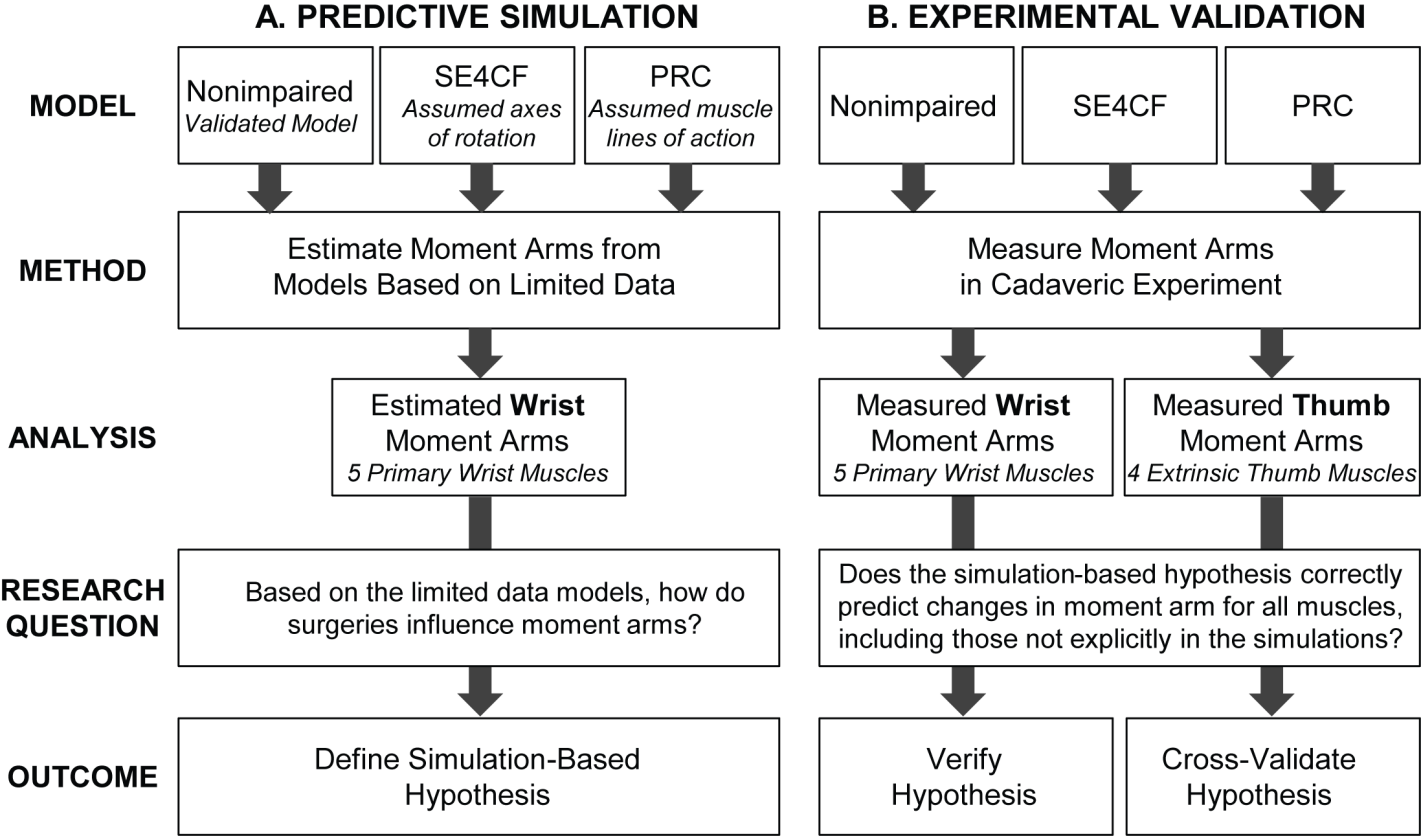
Study Design. Flowchart describing (A) predictive simulations to formulate a hypothesis regarding how muscle moment arms change following surgical salvage procedures and (B) the cadaveric experiment used to validate the simulation-based hypothesis.

To understand to what extent models based on limited quantitative data could predict moment arms following surgical salvage procedures, predictive simulations were performed using models of PRC and SE4CF wrists that were based on extremely limited data. From these predictive simulations, a hypothesis was formulated regarding how these surgeries influence moment arms (Fig 2A), and this hypothesis was consequently tested through a cadaveric experiment (Fig 2B). Importantly, the simulation-based hypothesis was based on analysis of only the primary wrist muscles, while the cadaveric experiment measured the moment arms of the primary wrist muscles as well as the extrinsic thumb muscles. The experimental data describing the moment arms of the primary wrist muscles was used to assess the validity of our simulation-based hypothesis, while the experimental data describing the moment arms of the extrinsic thumb muscles was used to evaluate whether our simulation-based hypothesis was generalizable to all muscles crossing the wrist.

### Musculoskeletal Modeling to Predict Moment Arms

The musculoskeletal models were developed in SIMM (Musculographics Inc.; [51]) by adapting a validated model of the nonimpaired wrist [52]. All models included bone geometry, joint kinematics, and muscle-tendon paths for the five primary wrist muscles (Fig 1B). The surgical models were based on the limited quantitative data available in the literature. The modeled geometric changes and axes of rotation have been described in detail previously [53], while the muscle-tendon path models have not been previously reported. For clarity, all modeling changes are described below.

**Fig 3:**
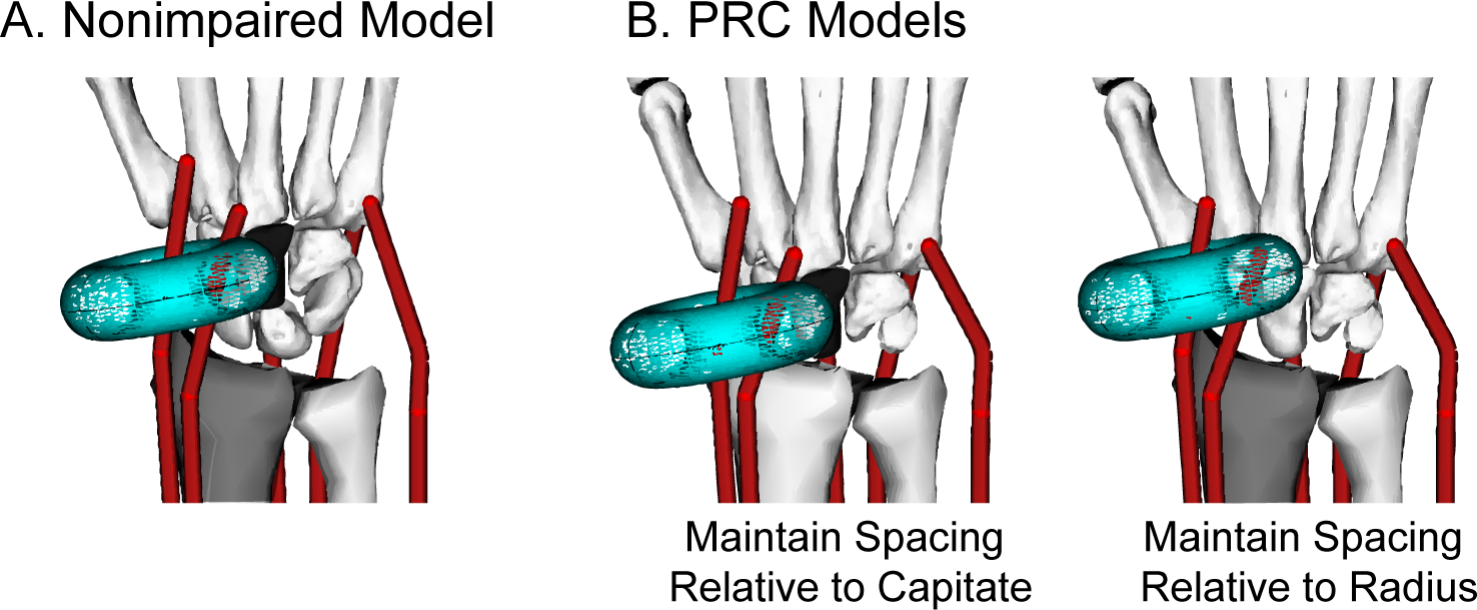
Muscle-Tendon Path Assumptions for the PRC Model. Illustration of a wrapping surface in (A) the nonimpaired and (B) the PRC models. In the nonimpaired model, the location of the wrapping surfaces is defined relative to the proximal row. In the PRC models, the location of the wrapping surfaces was redefined relative to the capitate by either maintaining the distance between the wrapping surface and capitate or maintaining the distance between the wrapping surface and radius. Shading of the capitate (black) and radius (gray) provides a visual reference to compare the location of the wrapping surface between the nonimpaired and PRC models. The torus shaped wrapping surface (shown for the ECRL) is representative of the wrapping surfaces implemented for each wrist muscle.

PRC was modeled using geometric changes described in the surgical literature, reported axes of rotation, and assumed muscle-tendon paths. The geometric changes involved removing the proximal row of carpal bones and translating the distal row and hand to establish an interface between the radius and capitate (Fig 1A). Axes of rotation were implemented based on the only study reporting wrist kinematics following PRC [12]. Muscle-tendon paths were re-defined because in the nonimpaired model the muscle-tendon wrapping surfaces were defined explicitly as a function of the proximal row (Fig 3A). After removing the proximal row in the PRC model, two methods were implemented to re-define muscle wrapping at the wrist. The first translated the wrapping surfaces with the proximal row, thereby maintaining the nonimpaired distance between the wrapping surface and the capitate (Fig 3B, left). The second did not translate the wrapping surfaces with the proximal row, thereby maintaining the nonimpaired distance between the wrapping surface and the radius (Fig 3B, right).

**Fig 4:**
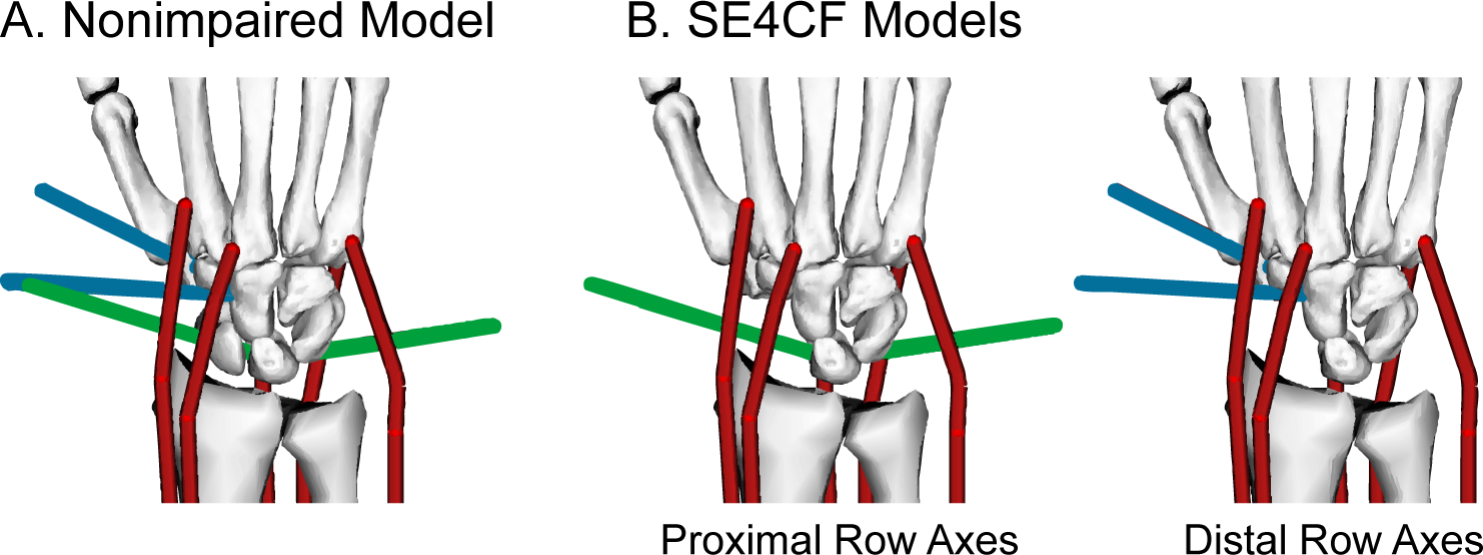
Axes of Rotation Assumption for the SE4CF Model. Illustration of the axes of rotation in the (A) nonimpaired and (B) SE4CF models. In the nonimpaired model, flexion-extension and radial-ulnar deviation axes of rotation separately define the motion of the proximal row relative to the radius (green axes) and the distal row relative to the proximal row (blue axes). In the SE4CF models, the motion of the fused carpal bones was defined using either the proximal row or distal row axes of rotation.

SE4CF was modeled using geometric changes described in the surgical literature, assumed axes of rotation, and muscle-tendon paths equivalent to those of the nonimpaired wrist. The geometric changes involved removing the scaphoid and fusing the lunate, capitate, hamate, and triquetrum using a weld joint (Fig 1A). Axes of rotation following SE4CF have not been reported; therefore, we simulated two different sets of axes of rotation, using assumptions based on the nonimpaired axes of rotation (Fig 4A) that have been previously described [53]. Briefly, the first set of axes of rotation assumed that SE4CF does not disturb the motion of the lunate relative to radius, thereby preserving the axes of rotation of the nonimpaired proximal row (Fig 4B, right). The second set of axes assumed that SE4CF does not disturb the motion of the capitate relative to the radius, thereby preserving the axes of the nonimpaired distal row (Fig 4B, left). Muscle-tendon paths were defined equivalent to those in the nonimpaired model because all of the bone geometry used to define the muscle-tendon paths remained in an equivalent position relative to the radius after simulating SE4CF.

For the two PRC models (each with different muscle-tendon path definitions) and the two SE4CF models (each with different joint axes of rotation), moment arms were calculated for each wrist degree of freedom (flexion-extension and radial-ulnar deviation) for the primary wrist muscles at a neutral position using two methods: (i) the partial velocity method [51] and (ii) the perpendicular distance between the muscle line of action and the joint axis of rotation [54]. This resulted in four moment arm estimates for each muscle, procedure, and degree of freedom. Similarly, moment arms were calculated for the nonimpaired model using both moment arm calculation methods. Average moment arms across all modeling methods were then calculated for the nonimpaired, PRC, and SE4CF conditions. To predict how moment arms change following salvage procedures, the percent change in moment arm between the surgically salvaged and nonimpaired models were examined.

### Cadaveric Experiment to Measure Moment Arms

The moment arm experiment was designed to measure the muscle moment arms of the primary wrist and extrinsic thumb muscles. The data describing the primary wrist muscle moment arms has been previously described [47]. Briefly, muscle moment arms for the nonimpaired and surgically salvaged wrists were measured in eight unmatched, fresh-frozen cadaver upper extremities (four male, four female; avg. age 62.3 ± 8.9 years, range 44 to 73 years), using the tendon excursion method [54]. In accordance with the policies of the institutions at which this research was conducted, the cadaveric experiment was exempt from IRB approval. Cadaveric specimens were obtained from Medcure, Inc. (Portland, OR) a tissue bank that is fully accredited through the American Association of Tissue Banks (AATB) and ensures that all specimens are obtained (i) with the appropriate informed consent of donor or donor's next-of-kin and (ii) in compliance with the Uniform Anatomical Gift Act and all other local, state, and federal laws and regulations governing the recovery and distribution of anatomical specimens.

In each specimen, data were collected sequentially for three conditions: nonimpaired, SE4CF, and PRC. To simulate SE4CF, the scaphoid was excised and Kirschner wires were used to fuse the lunate, capitate, hamate, and triquetrum. To simulate PRC, the Kirschner wires were removed and the remaining proximal carpal bones (lunate and triquetrum) were excised. Soft tissue and the finger extensor tendons were imbricated to establish an interface between the radius and capitate. Board-certified hand surgeons performed the surgical procedures.

Moment arms, defined as the derivative of tendon excursion with respect to joint angle, were determined for each specimen, condition, muscle, and degree of freedom. Tendon excursions were simultaneously recorded from the five primary wrist muscles and the four extrinsic thumb muscles using potentiometers (Model 3543s, Bourns Inc.). Joint angles were calculated as the angle between the long axes of the third metacarpal and radius, which was measured by a motion capture system (Optotrak Certus, Northern Digital Inc.). All data was collected during passive, planar wrist motion for both flexion-extension and radial-ulnar deviation. Data were smoothed by fitting fourth order polynomials to the moment arm versus joint angle curves for each combination of specimen, muscle, surgical condition, and degree of freedom.

Statistically significant differences between the nonimpaired, PRC, and SE4CF moment arm versus joint angle curves measured in the experiment were determined using mixed effects models, including condition and joint angle as fixed factors and specimen as a random factor. A significance level of p<0.05 was used for all tests. Multiple comparisons with a Tukey correction were used when the F-test of the ANOVA was significant.

### Evaluation of Simulation-Based Hypothesis

The simulation-based hypothesis was evaluated in two stages that utilized the experimental data from the primary wrist and extrinsic thumb muscles, respectively. In the first stage, the muscle moment arms for the primary wrist muscles were examined to determine if the trends predicted by the models were identified in the experiment. Also in this stage, the moment arms predicted by the model were directly compared to those measured in the experiment. For this direct comparison, moment arms at a neutral position were calculated from the experimental data because the simulation-based hypothesis was based on estimation of moment arms in a neutral position. Moment arms at a neutral position were defined as the average muscle moment arm for each muscle, surgical condition, and degree of freedom at zero degrees flexion-extension and zero degrees radial-ulnar deviation. In the second stage, the muscle moment arms for the extrinsic thumb muscles were examined to determine if the trends predicted by the simulation-based hypothesis were generalizable to other muscles crossing the wrist that were not explicitly modeled. This use of the extrinsic thumb muscle data was a form of cross-validation.

## Results

**Fig 5:**
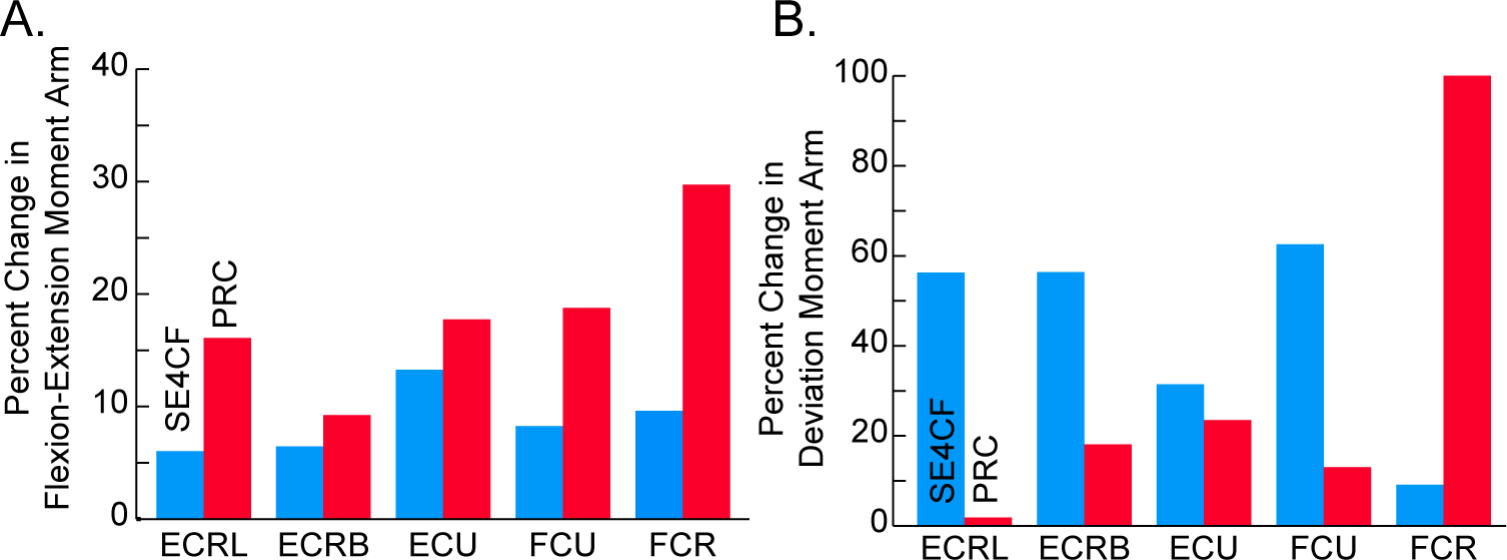
Percent Change in Model Predicted Muscle Moment Arms. Average percent change in (A) flexion-extension and (B) radial-ulnar deviation moment arms for the SE4CF (blue) versus the PRC (red) models relative to the nonimpaired model. Note that the y-axis scales are different between the two panels.

The musculoskeletal models suggest that PRC primarily alters flexion-extension moment arms, while SE4CF primarily alters radial-ulnar deviation moment arms. Specifically, when compared to the moment arms estimated by the nonimpaired model, the flexion-extension moment arms estimated by the PRC models demonstrated larger changes in magnitude than those estimated by the SE4CF models for all five primary wrist muscles (cf., Fig 5A, red bars greater than blue bars for all muscles). In contrast, the radial-ulnar deviation moment arms estimated by the PRC models demonstrated smaller changes in magnitude than those estimated by the SE4CF models for four out of five muscles (cf., Fig 5B, red bars less than blue bars for all muscles, except FCR). The specific moment arm values predicted by the models are summarized in Table 1.

**Table 1:** Wrist Muscle Moment Arms Predicted by the Models^#^.

Muscle	Flexion-Extension			Radial-Ulnar Deviation		
	Nonimpaired	SE4CF	PRC	Nonimpaired	SE4CF	PRC
**FCR**	-1.37 (0.17)	-1.49 (0.35)	-1.76 (0.19)	-0.57 (0.25)	-0.56 (1.47)	0.00 (0.19)
**FCU**	-1.46 (0.04)	-1.58 (0.26)	-1.73 (0.03)	1.96 (0.31)	0.73 (1.71)	1.68 (0.21)
**ECRB**	1.26 (0.10)	1.18 (0.18)	1.14 (0.31)	-1.16 (0.24)	-0.50 (0.90)	-0.93 (0.14)
**ECRL**	0.93 (0.05)	0.88 (0.17)	0.78 (0.15)	-2.13 (0.01)	-0.93 (0.50)	-2.16 (0.40)
**ECU**	0.70 (0.09)	0.60 (0.70)	0.57 (0.05)	2.46 (0.06)	1.68 (0.94)	3.03 (0.46)

^#^Moment arms reported in centimeters at a neutral wrist posture. Positive values indicate extension and ulnar deviation. Values in parentheses represent one standard deviation, thereby denoting variability due to modeling technique.

**Fig 6:**
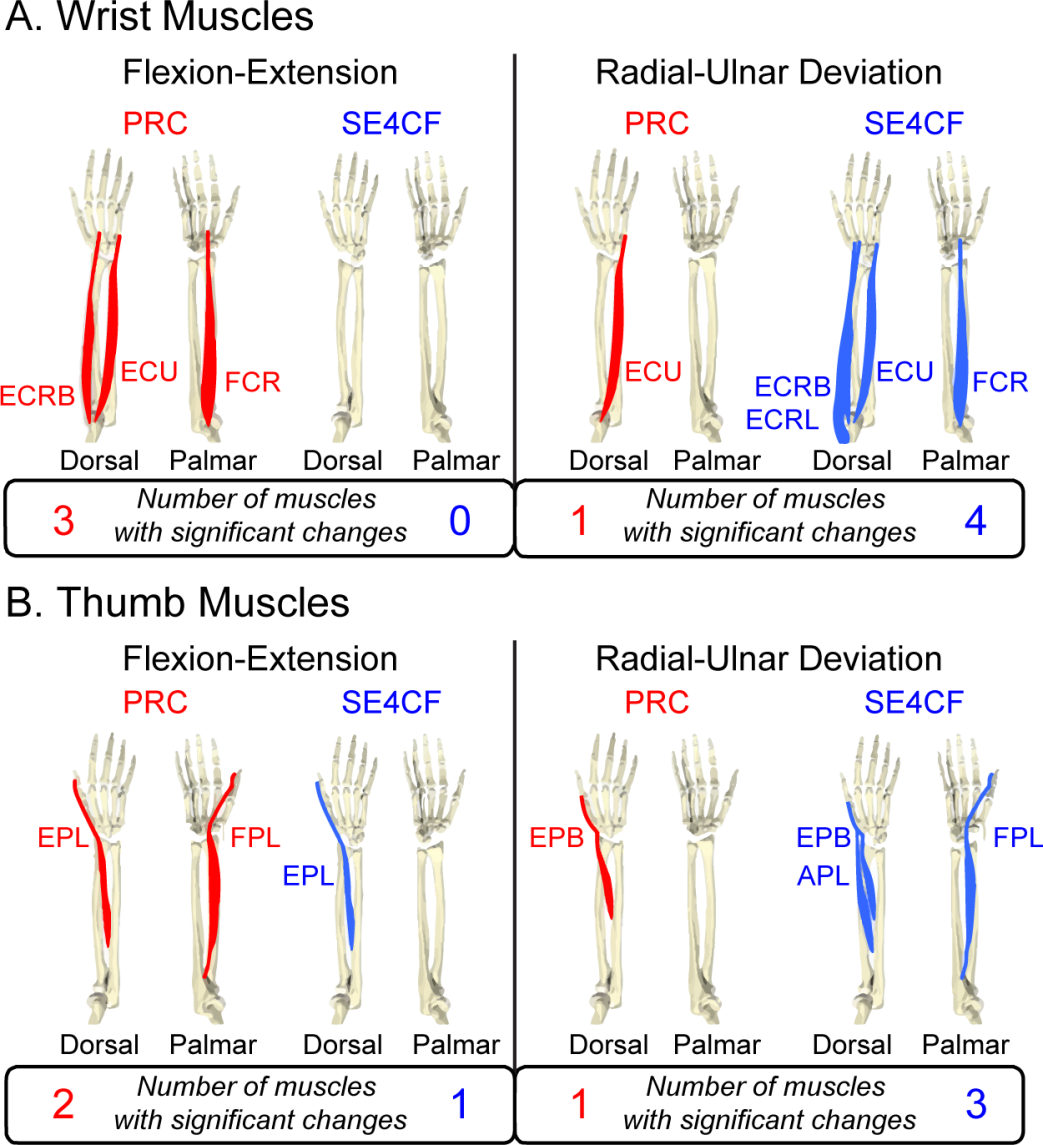
Significant Differences in Experimentally Measured Muscle Moment Arms. Depicted muscles indicate statistically significant differences (p<0.05) between the nonimpaired and surgically salvaged moment arm versus joint angle curves, as measured experimentally. (A) Wrist and (B) thumb muscles are displayed separately to illustrate similar trends in how PRC (red) and SE4CF (blue) influence flexion-extension moment arms (left) and radial-ulnar deviation moment arms (right) across the two distinct muscle groups.

The experimental results for the primary wrist muscles support the hypothesis that PRC primarily alters flexion-extension moment arms, while SE4CF primarily alters radial-ulnar deviation moment arms. When comparing the nonimpaired and surgically altered flexion-extension moment arms, more muscles demonstrated statistically significant changes following PRC than SE4CF (c.f., Fig 6A, left, three muscle significantly altered following PRC versus zero muscles following SE4CF). Alternatively, when comparing radial-ulnar deviation moment arms, fewer muscles demonstrated statistically significant changes following PRC than SE4CF (c.f., Fig 6A, right, one muscle significantly altered following PRC versus four muscles following SE4CF). The specific wrist muscle moment arm values measured experimentally are summarized in Table 2.

**Table 2:** Primary Wrist Muscles: Experimentally Measured Wrist Muscle Moment Arms^#^.

Muscle	Flexion-Extension			Radial-Ulnar Deviation		
	Nonimpaired	SE4CF	PRC	Nonimpaired	SE4CF	PRC
**FCR**	-1.55 (0.47)	-1.32 (0.44)	-1.04 (0.34)*	-0.85 (0.35)	-1.20 (0.59)*	-0.69 (0.35)
**FCU**	-1.47 (0.27)	-1.51 (0.22)	-1.40 (0.31)	1.81 (0.61)	1.56 (0.71)	1.67 (0.83)
**ECRB**	1.25 (0.33)	1.41 (0.39)	1.36 (0.36)*	-1.75 (0.85)	-2.14 (0.49)*	-1.31 (0.56)
**ECRL**	0.93 (0.35)	0.99 (0.37)	1.11 (0.23)	-2.20 (0.34)	-2.63 (0.32)*	-1.81 (0.15)
**ECU**	0.63 (0.10)	0.57 (0.30)	0.04 (0.17)*	1.94 (0.40)	1.56 (0.53)*	0.90 (0.86)*
**Total:**	---	0	3	---	4	1

^#^Moment arms reported in centimeters at a neutral wrist posture. Positive values indicate extension and ulnar deviation. Values in parentheses represent one standard deviation, thereby denoting variability due to specimen. Total indicates number of muscles with significantly altered moment arms following the given surgery.

*Denotes significant difference (p<0.05) between nonimpaired and surgically salvaged moment arm versus joint angle curves.

The experimental results for the extrinsic thumb muscles demonstrate that the simulation-based hypothesis is generalizable. Similar trends for both flexion-extension moment arms (c.f., Fig 6B, left, two muscle significantly altered following PRC versus one muscles following SE4CF) and radial-ulnar deviation moment arms (c.f., Fig 6B, right, one muscle significantly altered following PRC versus three muscles following SE4CF) were observed for the extrinsic thumb muscles. The specific thumb muscle moment arm values measured experimentally are summarized in Table 3.

**Table 3:** Extrinsic Thumb Muscles: Experimentally Measured Wrist Muscle Moment Arms^#^.

Muscle	Flexion-Extension			Radial-Ulnar Deviation		
	Nonimpaired	SE4CF	PRC	Nonimpaired	SE4CF	PRC
**FPL**	-1.46 (0.30)	-1.75 (0.43)	-1.14 (0.37)*	-0.71 (0.26)	-1.16 (0.30)*	-0.61 (0.48)
**EPL**	0.73 (0.36)	0.85 (0.33)*	1.03 (0.41)*	-1.56 (0.56)	-1.76 (0.48)	-0.88 (0.81)
**APL**	-0.76 (0.31)	-0.89 (0.52)	-0.72 (0.46)	-2.05 (0.41)	-2.76 (0.26)*	-1.35 (1.03)
**EPB**	-0.55 (0.24)	-0.55 (0.32)	NA	-2.27 (0.73)	-2.78 (0.29)*	-0.81 (0.81)*
**Total:**	---	1	2	---	3	1

^#^Moment arms reported in centimeters at a neutral wrist posture. Positive values indicate extension and ulnar deviation. Values in parentheses represent one standard deviation, thereby denoting variability due to specimen. NA indicates that the condition was not analyzed due to a paucity of data. Total indicates number of muscles with significantly altered moment arms following the given surgery.

*Denotes significant difference (p<0.05) between nonimpaired and surgically salvaged moment arm versus joint angle curves.

## Discussion

This study demonstrates that musculoskeletal models, even if they are based on extremely limited amounts of quantitative data, can provide important insights. The moment arms estimated by the models and measured in the cadaveric experiment both indicate that a critical difference between PRC and SE4CF is how they alter radial-ulnar deviation versus flexion-extension moment arms at the wrist. Although numerous studies have examined post-operative differences between PRC and SE4CF [55–58], the biomechanical factors contributing to reported differences are not known. In particular, both PRC and SE4CF are known to cause functional impairments in wrist range of motion and grip strength [57]. Understanding how separately altering the wrist’s degrees of freedom influences these functional impairments may be instrumental in delineating post-operative differences between PRC and SE4CF.

This study also demonstrates that musculoskeletal models can be used to inform the design of experiments. Traditionally, surgically altered moment arms are examined through cadaveric experiments by performing a muscle-by-muscle comparison of whether changes in moment arm due to a given surgery are statistically significant. However, incremental changes in individual muscle moment arms for multiple muscles at a given joint are difficult to interpret. In this study, we built upon these traditional analyses by using musculoskeletal models to learn about the system before performing an experiment. The need to synthesize and interpret our modeling analyses directly informed our hypothesis, which then guided our experimental design. Specifically, examining global changes in muscle actions, including changes to groups of muscles and differences between degrees of freedom, led to the decision to examine changes in flexion-extension versus radial-ulnar deviation moment arms in the nonimpaired, SE4CF, and PRC wrists.

**Fig 7:**
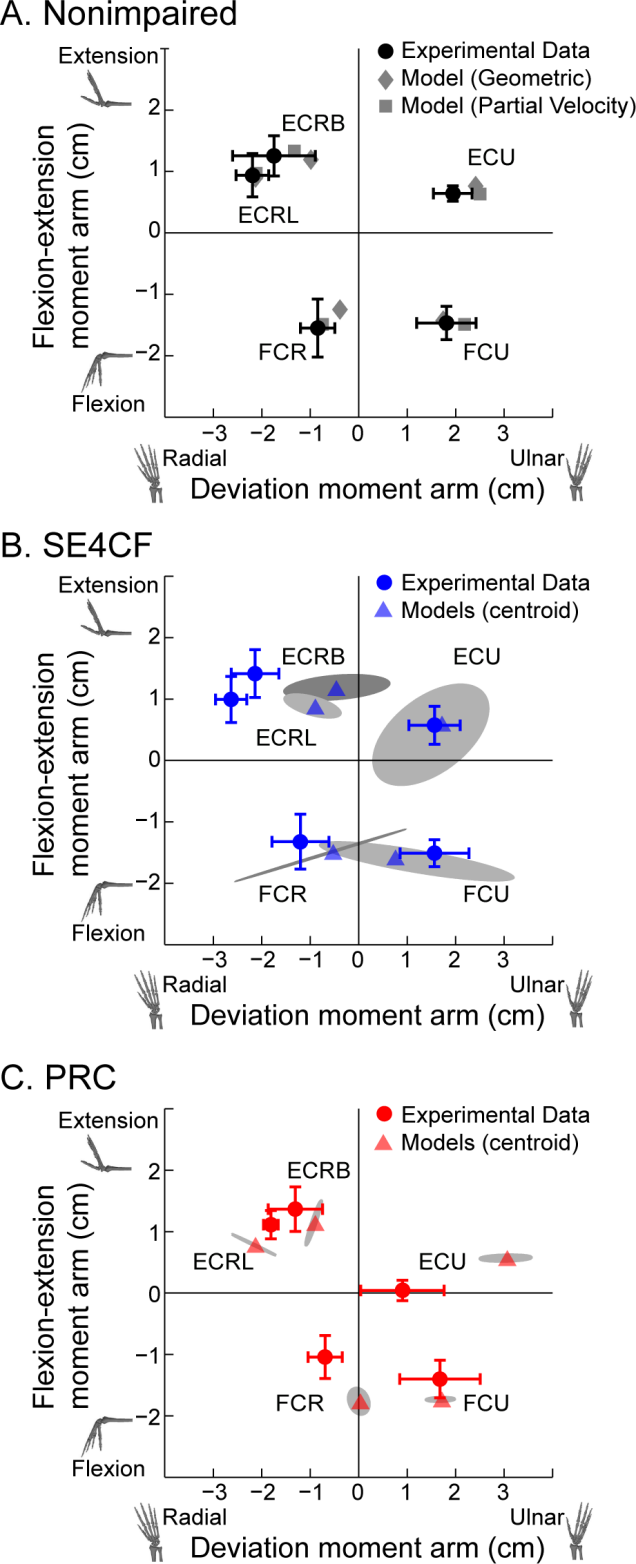
Model Predicted and Experimentally Measured Moment Arm Comparison. Comparison of moment arms predicted by the models (non-circular shapes) to moment arms measured in the experiment (filled circles) for the (A) nonimpaired, (B) SE4CF, and (C) PRC wrists. In each graph, flexion-extension moment arms are plotted as a function of radial-ulnar deviation moment arms. Error bars represent one standard deviation. In (B) and (C), the model data (triangles) represent the centroid of the moment arms predicted by the surgical models, and the shaded regions are the minimum area ellipses that enclose the predicted values, thereby representing the spread of the model predictions. All values are moment arms at a neutral wrist posture.

The simulation-based hypothesis was robust because it accurately predicted clinically relevant, global changes in muscle moment arms for both the primary wrist and extrinsic thumb muscles. However, we were unable to exactly predict the values of moment arms for specific muscles through surgical simulations (Fig 7). To further improve the ability of musculoskeletal models to exactly predict moment arms, techniques must be developed to accurately guide modeling decisions when data is limited or not yet available. For example, the SE4CF model, which was based on assumed axes of rotation, closely predicted flexion-extension moment arms, but not radial-ulnar deviation moment arms for the primary wrist muscles (Fig 7B, flexion-extension moment arms predicted by models within one standard deviation of those measured experimentally for all five muscles, versus only one muscle for radial-ulnar deviation moment arms). This suggests that the assumed radial-ulnar deviation axes of rotation in the SE4CF models do not accurately capture the kinematics of the SE4CF wrist. Techniques, such as utilizing motion predictions based on contact modeling of the surgically altered joint interface, may lead to more accurate estimation and implementation of unknown axes of rotation in surgical models.

In contrast, the PRC model, which was based on assumed muscle lines of action, closely predicted both flexion-extension and radial-ulnar deviation moment arms for two of five primary wrist muscles (Fig 7C, flexion-extension and radial-ulnar deviation moment arms predicted by models within one standard deviation of those measured experimentally for ECRB and FCU). This suggests that the assumed position of the wrapping surfaces in the PRC models more accurately captured the muscle lines of action for these two muscles than the other three. Although magnetic resonance imaging techniques exist to estimate muscle moment arms, these methods require extensive data collection and analysis [59, 60]. Improving methods for quickly capturing the anatomical constraints dictating how muscle lines of action are guided by bones and soft tissue may lead to more accurate and efficient implementation of unknown muscle paths in surgical models.

This study illustrates that biomechanical models based on extremely limited data sets provide novel insights that can be used to guide the design of experiments and test the predictive limits of current computer simulation techniques. Challenges remain before we can predict the exact values of moment arms for a specific surgical candidate through surgical simulations. However, models are valuable tools when examining of how the geometric changes imposed by orthopaedic surgery impact clinical outcomes.
